# Preparing for the worst: opportunities to prevent trans-boundary disease transmission in Uganda: a case study

**DOI:** 10.11604/pamj.supp.2022.41.1.31195

**Published:** 2022-03-23

**Authors:** Alex Riolexus Ario, Daniel Kadobera, Benon Kwesiga, Stephen Ndugwa Kabwama, Lilian Bulage

**Affiliations:** 1 Uganda National Institute of Public Health, P.O Box 7272, Kampala, Uganda,; 2 Ministry of Health, Kampala, Uganda,; 3 College of Health Sciences, Makerere University School of Public Health, Kampala, Uganda

**Keywords:** Case Study, Ebola Virus Disease, Cross-border transmission, Uganda

## Abstract

On August 1^st^, 2018, the Ministry of Health of the Democratic Republic of Congo (DRC) declared its tenth Ebola Virus Disease (EVD) outbreak in history, affecting North Kivu and Ituri provinces. The outbreak response was complicated due to insecurity and armed conflict in the region, and over the next 19 months, thousands of cases and deaths would occur, making this the world´s second-largest outbreak of EVD to date. On 4 August 2018, the Uganda Ministry of Health (MoH) activated the national coordination mechanisms for public health emergencies. The National Rapid Response Team (NRRT) immediately mobilized and embarked on a preparedness assessment and risk mapping to inform the country´s EVD response plan. This case study describes the events that transpired from declaration, activation of the coordination mechanisms, preparedness and response to EVD. The case study is meant to teach rapid responders, Incident Management Team members and the National and District Task Forces on how to prepare and respond to such outbreaks.

## How to use this case study

**How to use this case study:** case studies in applied epidemiology allow students to practice applying epidemiologic skills in the classroom to address real-world public health problems. The case studies are used as a vital component of an applied epidemiology curriculum, rather than as stand-alone tools. They are ideally suited to reinforce principles and skills already covered in a lecture or in background reading. This case study has a facilitator guide and a participant guide. Each facilitator should review the Facilitator Guide, gain familiarity with the outbreak and investigation on which the case study is based, review the epidemiologic principles being taught, and think of examples in the facilitator´s own experience to further illustrate the points. Ideally, participants receive the case study one part at a time during the case study session. However, if the case study is distributed in whole, participants should be asked not to look ahead.

During the case study session, one or two instructors facilitate the case study for 8 to 20 students in a classroom or conference room. The facilitator should hand out Part I and direct a participant to read one paragraph out loud, then progressing around the room and giving each participant a chance to read. Reading out loud and in turns has two advantages. First, all participants engage in the process and overcome any inhibitions by having her/his voice heard. Second, it keeps all the participants progressing through the case study at the same speed.

After a participant reads a question, the facilitator will direct participants to answer the question and perform calculations, construct graphs, or engage in a discussion of the answer. Sometimes, the facilitator can split the class to play different roles or take different sides in answering the question. As a result, participants learn from each other, not just from the facilitator.

After the questions have been answered, the facilitator hands out the next part. At the end of the case study, the facilitator should direct a participant to once again read the objectives on page 1 to review and ensure that the objectives have been met.

**Prerequisites**: for this case study, participants should have received instruction or conducted readings in: *National Multi-Hazard Preparedness and Response Plan for Public Health Threats and Emergencies in Uganda*; *WHO Framework for a Public Health Emergency Operations Centre*.

**Target audience**: trainees in the Field Epidemiology Training Program / Public Health Fellowship Program, other Field Epidemiology and Laboratory Training Programs (FELTPs), public health students, public health workers who may participate in rapid needs assessments, and others who are interested in this topic.

**Level of case study**: advanced

**Time required**: provide expected duration (e.g., approximately 4 hours)

**Language**: English

## Case study material

- Download the case study student guide (PDF - 910 KB)
- Request the case study facilitator guide
